# Beyond the method change in clinical practice: evaluation of insulin-like growth factor I assay

**DOI:** 10.1515/almed-2021-0069

**Published:** 2022-08-23

**Authors:** Paula Sienes Bailo, Marta Fabre Estremera, José Cuenca Alcocel, María Ángeles César Márquez

**Affiliations:** Department of Clinical Biochemistry, Miguel Servet University Hospital, Zaragoza, Spain; Department of Clinical Biochemistry, Miguel Servet University Hospital, Zaragoza, Spain; and Aragon Institute of Health Research (IIS Aragon), Zaragoza, Spain

**Keywords:** growth hormone, immunoassay, insulin-like growth factor I, method comparison

## Abstract

**Objectives:**

Insulin-like growth factor I (IGF-I) is the preferred biomarker for diagnosing and monitoring growth-related disorders but its serum quantification presents several difficulties since different IGF-I assays still leads to different IGF-I concentrations, especially when results are either above or below the normal range.

**Methods:**

We conducted a prospective study between November and December 2020 at a tertiary University Hospital with 212 serum samples to determine the analytical performance of the IGF-I assay on the Cobas e411 (Roche Diagnostics) and compare it with that of the Immulite 2000XPi (Siemens).

**Results:**

In this work, we report for the first time the existence of discrepancies between IGF-I levels measured by Immulite 2000XPi and Cobas e411. Deming regression model provided a slope of 1.570 (95% CI: 1.395–1.745) and an intercept of −58.591 (95% CI: −89.151 to −28.030), with R^2^=0.967 and average bias of +53.061 with overestimation of IGF-I. It was found that Cobas e411 provides abnormally high IGF-I concentrations, but further studies are required to elucidate the cause of the discrepancies.

**Conclusions:**

Our data can alert clinicians and laboratory professionals of this situation and avoid misinterpretation of increased IGF-I levels as a therapeutic failure rather than as a problem associated with this method change.

Insulin-like growth factor I (IGF-I) is a 70 amino acid polypeptide hormone mainly synthesized in the liver which acts as the principal peripheral mediator of growth hormone (GH) action. Its synthesis and secretion are directly regulated by GH, being the main peripheral marker of its action and the one that best correlates with the secretory state of GH in postnatal life. Many factors cause short-term fluctuations in GH secretory status including pulsatile release from the pituitary gland, circadian periodicity and sleep; however, these factors have minimal or no effect on IGF-I levels. This fact makes IGF-I the preferred biomarker for the diagnosis and monitoring of growth-related disorders, ranging from GH deficiency to excess [1, 2].

There are several immunoassays for the serum IGF-I quantification that have to face the challenges associated with its binding to transporter proteins, the long-term stability of calibration standards, the cross-reactivity with IGF-II and the development of reliable age- and sex-specific reference intervals (RIs). Assays are generally standardized against the World Health Organization (WHO) NIBSC 87/815 or against the WHO NIBSC IS 02/254, but although important advances have been made for its harmonization, IGF-I measurement with different assays still leads to different IGF-I concentrations, especially when the results are either above or below the normal range. This may be due to differences in the method design (competitive, immunometric or others), antibody specificity and affinity, protein binding interference, reference preparations and sensitivity [3, 4].

Therefore, some authors propose to transform IGF-I concentrations into standard deviation scores (SDS) with respect to age, sex and pubertal stage, if these values ​​are available, although the non-normality of the reference values ​​of IGF-I greatly complicates the calculation of this parameter, having to resort to the use of spreadsheets or specific programs for this purpose [2].

On the other hand, the International Federation of Clinical Chemistry and Laboratory Medicine (IFCC) and the Laboratory and Clinical Standards Institute (CLSI) recommend that each laboratory determine its own RIs [5]. The establishment of paediatric RIs is especially complicated given the additional ethical problems associated with obtaining samples from minor patients. At least, each laboratory should transfer and verify its population from manufacturers’ instructions or studies published in the literature. The similarity between the reference population and the population served by the laboratory is a crucial factor to take into consideration, especially in those parameters that depend on ethnicity, sex and age. Therefore, RIs should not be automatically extrapolated to other different settings [6].

Clinical laboratories are well aware of the different strategies to follow during the change of methods. The veracity of a method can be evaluated by following either the CLSI EP15-A2 or the CLSI EP09-A3 guidelines, both providing guidance on how to estimate the bias and concordance between assay methods using patient samples. It is recommended that any change in the method might be notified in the laboratory report in order to avoid misinterpretation with results obtained by other different methods. Differences between methods can be significant and lead to changes in the diagnosis, treatment initiation or long-term management of patients with GH-related disorders [3].

We report a recent situation in our laboratory. Due to a shift of public tender, we proceeded to change the IGF-I assay from an enzyme-labelled chemiluminescent immunometric assay (Immulite 2000XPi, Siemens Healthcare Diagnostics, UK) to an electrochemiluminescence immunoassay (Cobas e411, Hoffmann-La Roche, Switzerland). Prior to the change, we conducted an evaluation study and we accepted the change proposed. Laboratory reports included the new methodology and RIs.

Within months, we observed a suspiciously high proportion of samples with IGF-I concentrations above the age- and sex-specific RIs provided by the new manufacturer. Clinicians also observed discrepancies between IGF-I measurements by Cobas e411 and the clinical course of several patients. This has meant an increase of medical consultations and laboratory and imaging studies for the purpose of check patients’ status.

In order to determine if IGF-I results were measured correctly on Cobas e411, a prospective study was performed. Firstly, we checked this analyser and after re-evaluated Roche and Siemens IGF-I assays. During a month, 212 randomly selected serum samples from clinical laboratory routine patients of the Miguel Servet University Hospital (Zaragoza, Spain) were assessed. The vast majority of patients came from Paediatric and Endocrinology Department for monitoring different pathologies such as acromegaly, growth retardation or pituitary adenoma. 114/212 (53.8%) of the cases were females and the remaining 98/212 (46.2%) were males. The age range was 2–90 years. Samples were collected in Vacutainer serum tubes with separator gel (Becton, Dickinson and Company, NJ, USA) and centrifuged after clot formation. The samples were analysed each day in both assays. In both methods (Cobas e411 and Immulite 200XPi), the calibrators used were traceable to the WHO NIBSC IS 02/254 standard. Before carrying out the analyses each day, both equipment internal quality control at two concentration levels were processed to ensure day-to-day consistency of the analytical process. The quality objectives were based on meeting the Desirable Biological Variation Database specifications (Table 1) [7]. The control was accepted when the result was within the 2sd range of the mean supplied by the manufacturer. Detailed clinical information was retrieved from the electronic medical records of the patients. Deming regression and Bland Altman analysis were performed for method comparison data. Statistical analysis, calculations and graphical presentation were carried out using IBM SPSS Statistics 20.0 and XLSTAT 2021.4 (2021) statistical and data analysis tool.

**Table 1:** Total error, bias and imprecision of the two levels of internal quality control for the assays.

	Desirable Biological Variation Database specifications	Cobas e411		Immulite 2000XPi	
		IQC1	IQC2	IQC1	IQC2
**CV%**	4.70	4.07	3.59	2.33	2.65
**Bias%**	7.15	5.30	4.81	4.70	1.69
**TE%**	14.9	12.02	10.73	8.55	6.07

TE, total error; CV, coefficient of variation; IQC, internal quality control.

Deming regression and Bland-Altman plots are shown in Figure 1. IGF-I results from Cobas e411 and Immulite 2000XPi showed good correlation (R^2^ = 0.967) with a slope of 1.570 (95% confidence interval (CI): 1.395–1.745) and an intercept of −58.591 (95% CI: −89.151 to −28.030) (Figure 1A). IGF-I results measured by Cobas e411 were generally higher than those of Immulite 2000XPi. We observed a mean bias of 53.061 units. For higher concentrations, data were even more distant to each other (Figure 1B). As an illustrative example of these discrepancies, a 12 year-old female with growth hormone deficiency on rhGH treatment for the past five years is reported (Case 27). The IGF-I levels of this female between March 2017 and September 2019 were measured by Immulite 2000XPi. Since October 2019, Cobas e411 was used. At that time, the IGF-I levels were above the RI and did not reflect her clinical status. In November 2020, IGF-I was determined by both assays. Figure 2 summarizes the patient’s IGF-I levels.

**Figure 1:**
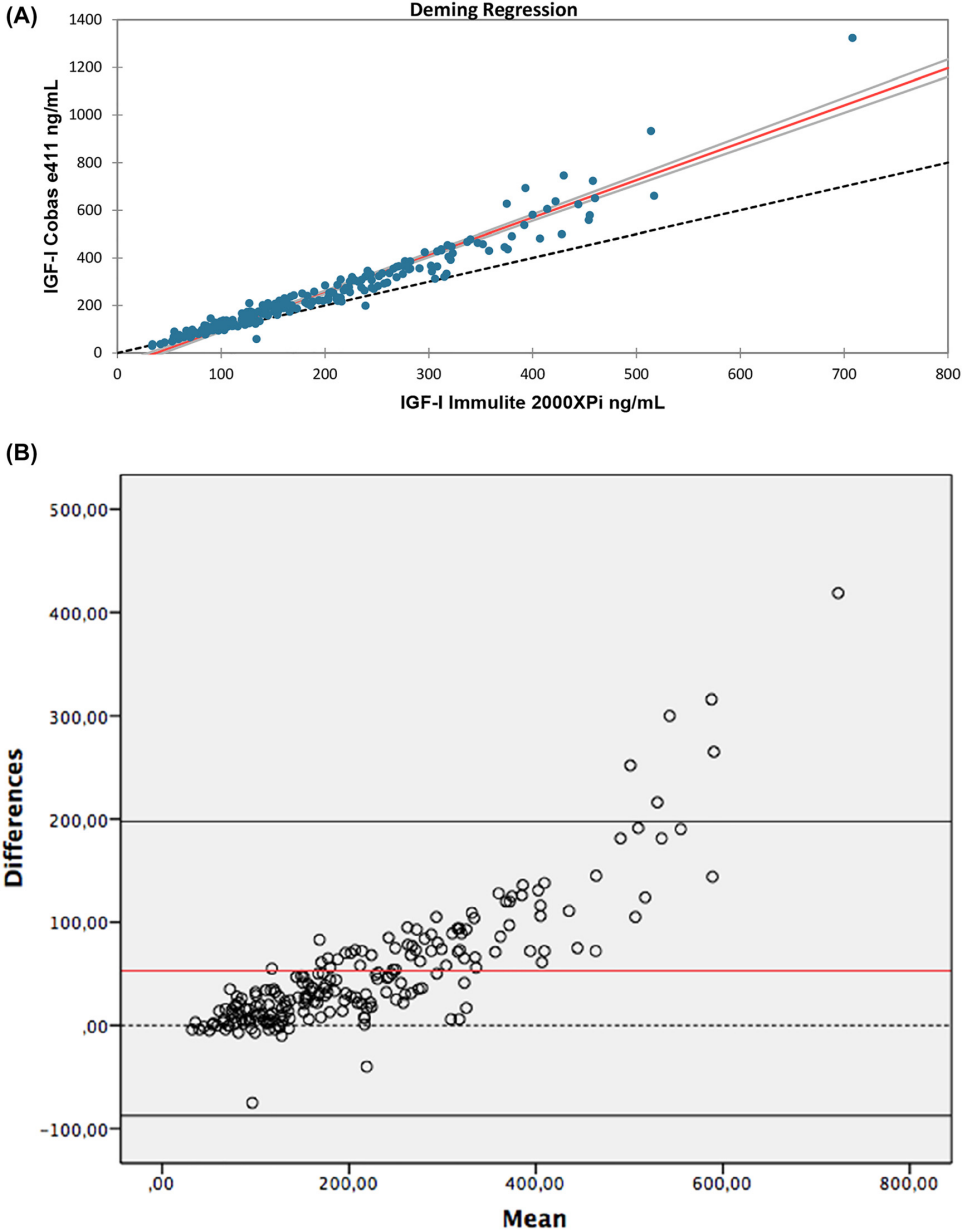
Method comparison data. (A) Deming regression. Scatterplot of IGF-1 values measured by each plataforms. Red line represents regression line. Blue dots represent IGF-1 values. (B) Bland-Alman plot. Horizontal black lines represent the mean of the difference between inmulite and Roche values, and the upper and lower limits of the 95% confidence interval. Dots represent IGF-1 values from each method. Red line represent the mean of the difference.

**Figure 2:**
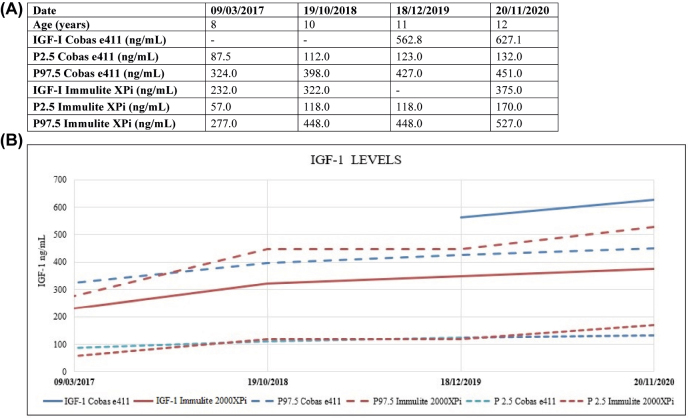
IGF-I levels. P2.5 and P97.5 percentiles correspond to the lower and upper limits of the reference intervals for each technique. (A) Table of IGF-I levels in the last 4 years. (B) Evolution of IGF-I levels in the last 4 years.

Mean circulating levels of IGF-I rise relatively slowly in childhood, but increase progressively during the prepubertal period and puberty, peaking at Tanner stage IV-V and then gradually decreasing [8]. The highest serum levels of IGF-I are found around 15 years old people [9]. Consequently, although it is possible to find high concentrations in this age group, it is not reasonable to find so many results above the RIs in patients without clinical symptoms. Furthermore, these abnormally high IGF-I levels measured by Cobas e411 are not justified by a lack of correlation between equipment (R^2^ = 0.967) or by common endogenous interferences such as haemolysis, lipemia, icterus or biotin. Nor does it appear to be due to exogenous factors such as treatment with rhGH. Studies carried out by the manufacturer rule out this possible interference. Furthermore, we have also found in our serie untreated patients with important differences between assays.

There is a growing literature on the evaluation and comparison of methods for the determination of common analytes such as haemoglobin A_1c_, cardiac and hepatic markers [10], [11], [12]. However, the literature on more specific analytes such as hormone markers is still scarce. In theory, the use of different analytic methods should not be a problem in clinical practice because commercial kits giving higher values should also have higher limits of normality, so patients should thus be consistently classified. However, this is indeed a problem that many laboratories are currently facing which makes it important to take this message into account and verify that those methods in which higher results of some magnitudes are obtained also have normality ranges that correlate with these values. In this report, we demonstrated that 35.5% of the IGF-I results measured by Cobas e411 were above the RIs compared to 16.5% of those measured with Immulite 2000XPi (Supplemental Data, Table S1). This has led to an increase in medical consultation and additional tests in our hospital. After this study, in according to Paediatrics Department, we decided return to daily work with Immulite 2000XPi.

This study has potential limitations. First, our laboratory adopted RIs from manufacturers. Secondly, our work group was unable to elucidate the cause of the discrepancies between IGF-I levels measured by Cobas e411 and the patient’s condition. Further studies are needed to find the cause of the discrepancies. Furthermore, kappa index would have added value to the study, but we did not have the necessary data to calculate. Despite these, our communication is designed to alert other professionals of this situation.

To our knowledge, this is the first study to describe higher IGF-I levels measured by Cobas e411 than by Immulite 2000XPi. Clinicians could interpret increased IGF-I levels as a therapeutic failure, although it could be a misinterpretation due to the analytical method. In addition, our data suggest that intercomparability studies showed a good correlation between both methods. But it is not enough to ensure the absence of problems in the change of methodology for some analytes, especially in the case of IGF-I used for monitoring treatments and with specific RIs by age and sex.

## Supplementary Material

Supplementary Material
