# Supplementary material for: Beyond the method change in clinical practice: evaluation of insulin-like growth factor I assay
Source: Adv Lab Med. 2022 Aug 23;3(4):397–401. doi: 10.1515/almed-2021-0069 (PMC10197437; doi:10.1515/almed-2021-0069)
Supplement: Supplementary file 1 — Supplementary Material [file j_almed-2021-0069_suppl.docx]

| **Demographic data** | | | **Cobas e411 (ng/mL)** | | **Immulite 2000XPi (ng/mL)** | | **Demographic data** | | | **Cobas e411 (ng/mL)** | | **Immulite 2000XPi (ng/mL)** | |
| --- | --- | --- | --- | --- | --- | --- | --- | --- | --- | --- | --- | --- | --- |
| **Case** | **Age (years)** | **Gender** | **IGF-I** | **P2.5-P97.5** | **IGF-I** | **P2.5-P97.5** | **Case** | **Age (years)** | **Gender** | **IGF-I** | **P2.5-P97.5** | **IGF-I** | **P2.5-P97.5** |
| 1 | 8 | M | 158.5 | 67.5-254.0 | 145.0 | 40.0-255.0 | 107 | 9 | M | 243.1 | 76.9-296.0 | 170.0 | 40.0-255.0 |
| 2 | 15 | M | 355.0 | 120.0-501.0 | 266.0 | 177.0-507.0 | 108 | 11 | M | 447.7 | 93.9-392.0 | 322.0 | 69.0-316.0 |
| 3 | 55 | F | 122.7 | 68.6-204.0 | 133.0 | 45.0-210.0 | 109 | 9 | F | 436.7 | 99.9-363.0 | 312.0 | 57.0-277.0 |
| 4 | 49 | M | 212.5 | 80.6-207.0 | 181.0 | 53.0-215.0 | 110 | 52 | M | 113.3 | 74.8-201.0 | 111.0 | 48.0-209.0 |
| 5 | 19 | M | 660.7 | 134.0-450.0 | 517.0 | 117.0-323.0 | 111 | 48 | M | 123.8 | 82.6-209.0 | 104.0 | 53.0-215.0 |
| 6 | 13 | F | 559.1 | 140.0-468.0 | 454.0 | 170.0-527.0 | 112 | 39 | M | 189.0 | 101.0-231.0 | 160.0 | 63.0-223.0 |
| 7 | 9 | F | 253.2 | 99.9-363.0 | 208.0 | 57.0-277.0 | 113 | 67 | F | 44.4 | 54.8-166.0 | 45.4 | 40.0-225.0 |
| 8 | 10 | M | 117.9 | 85.7-343.0 | 113.0 | 69.0-316.0 | 114 | 30 | M | 265.5 | 120.0-257.0 | 219.0 | 71.0-234.0 |
| 9 | 11 | M | 392.3 | 93.9-392.0 | 321.0 | 69.0-316.0 | 115 | 8 | F | 336.6 | 87.3-324.0 | 245.0 | 57.0-277.0 |
| 10 | 10 | F | 271.4 | 112.0-398.0 | 221.0 | 118.0-448.0 | 116 | 3 | F | 94.0 | 34.2-155.0 | 66.5 | 18.0-172.0 |
| 11 | 8 | M | 216.5 | 67.5-254.0 | 188.0 | 40.0-255.0 | 117 | 14 | M | 463.3 | 115.0-489.0 | 347.0 | 143.0-506.0 |
| 12 | 10 | M | 166.2 | 85.7-343.0 | 141.0 | 69.0-316.0 | 118 | 48 | F | 132.4 | 78.7-218.0 | 122.0 | 53.0-215.0 |
| 13 | 44 | F | 112.2 | 84.9-221.0 | 103.0 | 58.0-219.0 | 119 | 8 | M | 114.8 | 67.5-254.0 | 85.8 | 40.0-255.0 |
| 14 | 45 | F | 70.7 | 83.3-220.0 | 71.1 | 53.0-215.0 | 120 | 65 | M | 118.8 | 57.4-187.0 | 123.0 | 40.0-225.0 |
| 15 | 40 | M | 148.6 | 98.5-229.0 | 125.0 | 58.0-219.0 | 121 | 75 | F | 53.5 | 53.5-160.0 | 53.3 | 35.0-216.0 |
| 16 | 17 | M | 318.9 | 129.0-495.0 | 269.0 | 173.0-414.0 | 122 | 72 | F | 145.4 | 53.2-160.0 | 89.8 | 35.0-216.0 |
| 17 | 79 | M | 29.6 | 49.6-184.0 | 33.5 | 35.0-216.0 | 123 | 21 | F | 336.4 | 148.0-410.0 | 262.0 | 117.0-323.0 |
| 18 | 10 | M | 284.6 | 85.7-343.0 | 200.0 | 69.0-316.0 | 124 | 42 | M | 201.0 | 94.4-223.0 | 168.0 | 58.0-219.0 |
| 19 | 47 | F | 255.8 | 80.2-218.0 | 224.0 | 53.0-215.0 | 125 | 13 | M | 431.8 | 108.0-467.0 | 312.0 | 143.0-506.0 |
| 20 | 74 | F | 96.4 | 53.3-160.0 | 103.0 | 35.0-216.0 | 126 | 12 | F | 537.4 | 132.0-451.0 | 392.0 | 170.0-527.0 |
| 21 | 13 | M | 286.7 | 108.0-467.0 | 212.0 | 143.0-506.0 | 127 | 53 | F | 110.5 | 71.4-210.0 | 91.2 | 48.0-209.0 |
| 22 | 9 | M | 221.3 | 76.9-296.0 | 194.0 | 40.0-255.0 | 128 | 8 | F | 331.5 | 87.3-324.0 | 244.0 | 57.0-277.0 |
| 23 | 49 | M | 114.6 | 80.6-207.0 | 94.6 | 53.0-215.0 | 129 | 9 | F | 356.4 | 99.9-363.0 | 291.0 | 57.0-277.0 |
| 24 | 26 | F | 100.6 | 126.0-329.0 | 85.2 | 84.0-259.0 | 130 | 5 | M | 175.5 | 36.6-156.0 | 136.0 | 22.0-208.0 |
| 25 | 78 | M | 48.5 | 50.2-184.0 | 52.7 | 35.0-216.0 | 131 | 8 | F | 299.9 | 87.3-324.0 | 232.0 | 57.0-277.0 |
| 26 | 52 | M | 224.1 | 74.8-201.0 | 203.0 | 48.0-209.0 | 132 | 5 | F | 127.7 | 53.0-216.0 | 94.4 | 35.0-232.0 |
| 27 | 12 | F | 627.1 | 132.0-451.0 | 375.0 | 170.0-527.0 | 133 | 63 | F | 321.0 | 58.3-176.0 | 315.0 | 43.0-220.0 |
| 28 | 16 | M | 323.5 | 125.0-503.0 | 252.0 | 173.0-414.0 | 134 | 69 | M | 66.6 | 55.2-185.0 | 64.0 | 40.0-225.0 |
| 29 | 7 | M | 228.4 | 57.5-216.0 | 211.0 | 40.0-255.0 | 135 | 34 | M | 166.9 | 111.0-244.0 | 139.0 | 71.0-234.0 |
| 30 | 12 | M | 386.5 | 101.0-434.0 | 277.0 | 143.0-506.0 | 136 | 64 | M | 200.0 | 57.9-188.0 | 186.0 | 43.0-220.0 |
| 31 | 41 | F | 208.0 | 89.8-225.0 | 164.0 | 58.0-219.0 | 137 | 42 | F | 233.4 | 88.1-224.0 | 215.0 | 58.0-219.0 |
| 32 | 55 | F | 650.4 | 68.6-204.0 | 460.0 | 45.0-210.0 | 138 | 13 | M | 481.8 | 108.0-467.0 | 407.0 | 143.0-506.0 |
| 33 | 8 | M | 201.3 | 67.5-254.0 | 140.0 | 40.0-255.0 | 139 | 12 | F | 404.9 | 132.0-451.0 | 319.0 | 170.0-527.0 |
| 34 | 31 | F | 137.0 | 109.0-271.0 | 105.0 | 71.0-234.0 | 140 | 54 | F | 88.6 | 70.0-207.0 | 68.8 | 48.0-209.0 |
| 35 | 7 | F | 365.3 | 133.0-286.0 | 271.0 | 57.0-277.0 | 141 | 51 | F | 137.2 | 74.3-214.0 | 120.0 | 48.0-209.0 |
| 36 | 11 | M | 605.2 | 93.9-392.0 | 414.0 | 69.0-316.0 | 142 | 63 | F | 94.5 | 58.3-176.0 | 97.3 | 43.0-220.0 |
| 37 | 16 | F | 580.6 | 154.0-485.0 | 400.0 | 190.0-429.0 | 143 | 8 | F | 386.1 | 87.3-324.0 | 282.0 | 57.0-277.0 |
| 38 | 48 | F | 142.3 | 78.7-218.0 | 128.0 | 53.0-215.0 | 144 | 10 | F | 274.0 | 112.0-398.0 | 220.0 | 118.0-448.0 |
| 39 | 64 | F | 109.5 | 57.3-173.0 | 98.4 | 43.0-220.0 | 145 | 27 | F | 133.2 | 122.0-315.0 | 99.4 | 84.0-259.0 |
| 40 | 70 | M | 37.0 | 54.7-185.0 | 33.7 | 35.0-216.0 | 146 | 16 | M | 427.8 | 125.0-503.0 | 308.0 | 173.0-414.0 |
| 41 | 1 | M | 68.0 | 11.8-96.4 | 54.2 | <15.0-129.0 | 147 | 15 | M | 175.7 | 120.0-501.0 | 143.0 | 177.0-507.0 |
| 42 | 2 | M | 194.4 | 13.9-104.0 | 155.0 | <15.0-129.0 | 148 | 28 | F | 192.5 | 118.0-303.0 | 156.0 | 84.0-259.0 |
| 43 | 9 | F | 424.0 | 99.9-363.0 | 296.0 | 57.0-277.0 | 149 | 5 | M | 250.5 | 36.6-156.0 | 178.0 | 22.0-208.0 |
| 44 | 24 | F | 365.0 | 134.0-359.0 | 276.0 | 99.0-289.0 | 150 | 11 | F | 490.9 | 123.0-427.0 | 380.0 | 118.0-448.0 |
| 45 | 15 | F | 499.6 | 151.0-485.0 | 428.0 | 191.0-496.0 | 151 | 14 | M | 579.0 | 115.0-489.0 | 455.0 | 143.0-506.0 |
| 46 | 8 | M | 335.2 | 67.5-254.0 | 255.0 | 40.0-255.0 | 152 | 17 | M | 355.2 | 129.0-495.0 | 282.0 | 173.0-414.0 |
| 47 | 11 | F | 276.7 | 123.0-427.0 | 223.0 | 118.0-448.0 | 153 | 9 | F | 240.8 | 99.9-363.0 | 183.0 | 57.0-277.0 |
| 48 | 45 | F | 220.0 | 83.3-220.0 | 198.0 | 53.0-215.0 | 154 | 10 | M | 87.4 | 85.7-343.0 | 74.8 | 69.0-316.0 |
| 49 | 8 | F | 172.6 | 87.3-324.0 | 125.0 | 57.0-277.0 | 155 | 55 | M | 82.5 | 68.9-196.0 | 66.7 | 45.0-210.0 |
| 50 | 59 | M | 97.2 | 62.3-192.0 | 81.4 | 45.0-210.0 | 156 | 12 | F | 333.7 | 132.0-451.0 | 317.0 | 170.0-527.0 |
| 51 | 38 | M | 253.3 | 103.0-234.0 | 204.0 | 63.0-223.0 | 157 | 13 | F | 637.5 | 140.0-468.0 | 422.0 | 170.0-527.0 |
| 52 | 52 | F | 124.0 | 72.8-212.0 | 112.0 | 48.0-209.0 | 158 | 55 | M | 165.8 | 68.9-196.0 | 141.0 | 45.0-210.0 |
| 53 | 24 | M | 269.4 | 135.0-328.0 | 247.0 | 99.0-289.0 | 159 | 57 | M | 163.4 | 65.3-194.0 | 142.0 | 45.0-210.0 |
| 54 | 40 | F | 232.8 | 91.4-227.0 | 203.0 | 58.0-219.0 | 160 | 28 | M | 282.2 | 125.0-271.0 | 251.0 | 84.0-259.0 |
| 55 | 33 | F | 256.8 | 167.0-255.0 | 206.0 | 71.0-234.0 | 161 | 67 | F | 77.7 | 54.8-166.0 | 84.7 | 40.0-225.0 |
| 56 | 38 | F | 143.1 | 94.8-231.0 | 120.0 | 63.0-223.0 | 162 | 72 | F | 134.1 | 53.2-160.0 | 137.0 | 35.0-216.0 |
| 57 | 6 | F | 209.9 | 63.6-250.0 | 145.0 | 35.0-232.0 | 163 | 30 | M | 37.5 | 120.0-257.0 | 41.5 | 71.0-234.0 |
| 58 | 5 | M | 139.1 | 36.6-156.0 | 111.0 | 22.0-208.0 | 164 | 19 | M | 301.5 | 134.0-450.0 | 224.0 | 117.0-323.0 |
| 59 | 6 | F | 167.1 | 63.6-250.0 | 120.0 | 35.0-232.0 | 165 | 58 | M | 55.6 | 63.7-193.0 | 54.1 | 45.0-210.0 |
| 60 | 5 | M | 75.9 | 36.6-156.0 | 59.5 | 22.0-208.0 | 166 | 31 | M | 275.9 | 118.0-253.0 | 235.0 | 71.0-234.0 |
| 61 | 55 | F | 139.9 | 68.6-204.0 | 133.0 | 45.0-210.0 | 167 | 41 | F | 175 | 89.8-225.0 | 128.0 | 58.0-219.0 |
| 62 | 56 | F | 68.5 | 67.3-201.0 | 62.8 | 45.0-210.0 | 168 | 38 | F | 179.4 | 94.8-231.0 | 143.0 | 63.0-223.0 |
| 63 | 25 | F | 209.7 | 130.0-343.0 | 127.0 | 84.0-259.0 | 169 | 14 | M | 343.6 | 115.0-489.0 | 303.0 | 177.0-507.0 |
| 64 | 36 | F | 296.2 | 98.3-238.0 | 260.0 | 63.0-223.0 | 170 | 47 | M | 59.0 | 84.6-211.0 | 59.0 | 53.0-215.0 |
| 65 | 3 | F | 93.3 | 34.2-155.0 | 71.0 | 18.0-172.0 | 171 | 16 | F | 430.1 | 154.0-485.0 | 358.0 | 190.0-429.0 |
| 66 | 56 | M | 263.9 | 67.0-195.0 | 218.0 | 45.0-210.0 | 172 | 53 | F | 71.3 | 71.4-210.0 | 70.9 | 48.0-209.0 |
| 67 | 58 | F | 119.5 | 64.6-194.0 | 110.0 | 45.0-210.0 | 173 | 54 | F | 82.3 | 70.0-207.0 | 74.0 | 48.0-209.0 |
| 68 | 57 | F | 135.1 | 65.9-198.0 | 126.0 | 45.0-210.0 | 174 | 90 | F | 112.3 | 55.1-166.0 | 116.0 | 31.0-208.0 |
| 69 | 60 | F | 80.5 | 62.0-186.0 | 78.0 | 43.0-220.0 | 175 | 55 | F | 219.9 | 68.6-204.0 | 212.0 | 45.0-210.0 |
| 70 | 19 | M | 309.5 | 134.0-450.0 | 215.0 | 117.0-323.0 | 176 | 47 | F | 159.1 | 80.2-218.0 | 132.0 | 53.0-215.0 |
| 71 | 14 | M | 746.1 | 115.0-489.0 | 430.0 | 143.0-506.0 | 177 | 61 | F | 58.6 | 60.7-182.0 | 134.0 | 43.0-220.0 |
| 72 | 14 | F | 478.2 | 146.0-480.0 | 340.0 | 191.0-496.0 | 178 | 58 | M | 92.3 | 63.7-193.0 | 87.4 | 45.0-210.0 |
| 73 | 10 | F | 346.2 | 112.0-398.0 | 241.0 | 118.0-448.0 | 179 | 9 | F | 292.0 | 99.9-363.0 | 257.0 | 57.0-277.0 |
| 74 | 7 | F | 173.7 | 133.0-286.0 | 166.0 | 57.0-277.0 | 180 | 12 | M | 364.4 | 101.0-434.0 | 308.0 | 143.0-506.0 |
| 75 | 15 | F | 420.1 | 151.0-485.0 | 323.0 | 191.0-496.0 | 181 | 11 | F | 436.8 | 123.0-427.0 | 376.0 | 118.0-448.0 |
| 76 | 10 | M | 322.6 | 85.7-343.0 | 239.0 | 69.0-316.0 | 182 | 20 | F | 220.0 | 152.0-429.0 | 213.0 | 117.0-323.0 |
| 77 | 7 | M | 237.3 | 57.5-216.0 | 167.0 | 40.0-255.0 | 183 | 74 | M | 186.5 | 52.4-184.0 | 173.0 | 35.0-216.0 |
| 78 | 14 | M | 445.4 | 115.0-489.0 | 373.0 | 143.0-506.0 | 184 | 37 | F | 201.7 | 96.5-234.0 | 158.0 | 63.0-223.0 |
| 79 | 31 | F | 199.0 | 109.0-271.0 | 239.0 | 71.0-234.0 | 185 | 9 | M | 307.5 | 76.9-296.0 | 235.0 | 40.0-255.0 |
| 80 | 50 | F | 90.4 | 75.7-215.0 | 54.9 | 48.0-209.0 | 186 | 12 | F | 624.8 | 132.0-451.0 | 444.0 | 170.0-527.0 |
| 81 | 4 | M | 98.1 | 26.8-134.0 | 72.1 | 22.0-208.0 | 187 | 27 | M | 207.2 | 128.0-282.0 | 183.0 | 84.0-259.0 |
| 82 | 76 | F | 96.2 | 53.7-161.0 | 90.1 | 35.0-216.0 | 188 | 8 | F | 168.3 | 87.3-324.0 | 141.0 | 57.0-277.0 |
| 83 | 3 | M | 137.1 | 18.9-116.0 | 102.0 | <15.0-129.0 | 189 | 32 | M | 217.1 | 116.0-250.0 | 216.0 | 71.0-234.0 |
| 84 | 35 | M | 352.3 | 109.0-242.0 | 281.0 | 63.0-223.0 | 190 | 66 | F | 125.1 | 55.5-168.0 | 127.0 | 40.0-225.0 |
| 85 | 42 | F | 181.7 | 88.1-224.0 | 153.0 | 58.0-219.0 | 191 | 7 | F | 207.6 | 133.0-286.0 | 152.0 | 57.0-277.0 |
| 86 | 59 | F | 332.7 | 63.3-190.0 | 275.0 | 45.0-210.0 | 192 | 11 | F | 467.5 | 123.0-427.0 | 337.0 | 118.0-448.0 |
| 87 | 13 | F | 371.9 | 140.0-468.0 | 279.0 | 170.0-527.0 | 193 | 13 | M | 183.6 | 108.0-467.0 | 153.0 | 143.0-506.0 |
| 88 | 42 | M | 305.8 | 94.4-223.0 | 229.0 | 58.0-219.0 | 194 | 15 | M | 104.6 | 120.0-501.0 | 94.8 | 177.0-507.0 |
| 89 | 33 | F | 1324 | 167.0-255.0 | 708.0 | 71.0-234.0 | 195 | 77 | F | 95.5 | 54.0-162.0 | 92.0 | 35.0-216.0 |
| 90 | 71 | F | 130.6 | 53.3-161.0 | 127.0 | 35.0-216.0 | 196 | 15 | F | 312.4 | 151.0-485.0 | 306.0 | 191.0-496.0 |
| 91 | 8 | F | 192.3 | 87.3-324.0 | 142.0 | 57.0-277.0 | 197 | 45 | M | 116.5 | 88.5-216.0 | 113.0 | 53.0-215.0 |
| 92 | 7 | M | 258.5 | 57.5-216.0 | 189.6 | 40.0-255.0 | 198 | 16 | M | 457.9 | 125.0-503.0 | 352.0 | 173.0-414.0 |
| 93 | 15 | M | 932.9 | 120.0-501.0 | 514.0 | 177.0-507.0 | 199 | 72 | M | 87.9 | 53.6-184.0 | 82.0 | 35.0-216.0 |
| 94 | 14 | M | 693.4 | 115.0-489.0 | 393.0 | 143.0-506.0 | 200 | 52 | F | 114.2 | 72.8-212.0 | 110.0 | 48.0-209.0 |
| 95 | 60 | M | 173.5 | 61.1-191.0 | 151.0 | 43.0-220.0 | 201 | 35 | F | 192.8 | 100.0-242.0 | 161.0 | 63.0-223.0 |
| 96 | 48 | M | 170.9 | 82.6-209.0 | 130.0 | 53.0-215.0 | 202 | 44 | M | 123.6 | 90.5-218.0 | 124.0 | 58.0-219.0 |
| 97 | 11 | M | 319.3 | 93.9-392.0 | 226.0 | 69.0-316.0 | 203 | 33 | F | 263.0 | 167.0-255.0 | 238.0 | 71.0-234.0 |
| 98 | 25 | F | 179.3 | 130.0-343.0 | 142.0 | 84.0-259.0 | 204 | 4 | M | 116.4 | 26.8-134.0 | 83.9 | 22.0-208.0 |
| 99 | 22 | F | 197.4 | 143.0-39.2 | 147.0 | 99.0-289.0 | 205 | 64 | M | 66.2 | 57.9-188.0 | 70.3 | 43.0-220.0 |
| 100 | 47 | F | 88.2 | 80.2-218.0 | 69.0 | 53.0-215.0 | 206 | 8 | F | 367.7 | 87.3-324.0 | 302.0 | 57.0-277.0 |
| 101 | 23 | F | 81.3 | 138.0-375.0 | 69.3 | 99.0-289.0 | 207 | 45 | F | 160.5 | 83.3-220.0 | 154.0 | 53.0-215.0 |
| 102 | 8 | F | 220.2 | 87.3-324.0 | 156.0 | 57.0-277.0 | 208 | 3 | M | 275.1 | 18.9-116.0 | 245.0 | <15.0-129.0 |
| 103 | 18 | M | 453.8 | 132.0-476.0 | 318.0 | 173.0-414.0 | 209 | 13 | F | 723.4 | 140.0-468.0 | 458.0 | 170.0-527.0 |
| 104 | 9 | M | 234.1 | 76.9-296.0 | 212.0 | 40.0-255.0 | 210 | 76 | F | 110.7 | 53.7-161.0 | 106.0 | 35.0-216.0 |
| 105 | 14 | M | 230.7 | 115.0-489.0 | 161.0 | 143.0-506.0 | 211 | 38 | F | 177.2 | 94.8-231.0 | 155.0 | 63.0-223.0 |
| 106 | 66 | M | 141.9 | 56.8-186.0 | 123.0 | 40.0-225.0 | 212 | 33 | F | 87.8 | 167.0-255.0 | 86.2 | 71.0-234.0 |

**Table 1.** Comparison data.

IGF-I, insulin-like growth factor I; M, male; F, female; RI, reference intervals.
